# Investigation of epidemiological and clinical characteristics of people infected with SARS-CoV-2 during the second pandemic of COVID-19 in Chengdu, China

**DOI:** 10.3389/fpubh.2024.1394762

**Published:** 2024-05-01

**Authors:** Cheng Li, Tao Zhou, Peilin Zhang, Junning He, Yongfang Liu

**Affiliations:** ^1^Department of Infectious Diseases, The Third People’s Hospital of Chengdu, Chengdu, China; ^2^Department of Infectious Diseases, The Affiliated Hospital of North Sichuan Medical College, Nanchong, China

**Keywords:** re-infection, first infection, COVID-19, SARS-CoV-2, epidemiological characteristics, clinical characteristics

## Abstract

**Objective:**

This study investigated the epidemiological and clinical characteristics of Severe Acute Respiratory Syndrome Coronavirus 2 (SARS-CoV-2) infected patients during the second pandemic of COVID-19 (coronavirus disease of 2019) in Chengdu, China. Furthermore, the differences between first infection and re-infection cases were also compared and analyzed to provide evidence for better prevention and control of SARS-CoV-2 re-infection.

**Methods:**

An anonymous questionnaire survey was conducted using an online platform (wjx.cn) between May 20, 2023 to September 12, 2023.

**Results:**

This investigation included 62.94% females and 32.97% of them were 18–30 years old. Furthermore, 7.19–17.18% of the participants either did not receive vaccination at all or only received full vaccination, respectively. Moreover, 577 (57.64%) participants were exposed to cluster infection. The clinical manifestations of these patients were mainly mild to moderate; 78.18% of participants had a fever for 1–3 days, while 37.84% indicated a full course of disease for 4–6 days. In addition, 40.66% of the participants had re-infection and 72.97% indicated their first infection approximately five months before. The clinical symptoms of the first SARS-CoV-2 infection were moderate to severe, while re-infection indicated mild to moderate symptoms (the severity of symptoms other than diarrhea and conjunctival congestion had statistically significant differences) (*p* < 0.05). Moreover, 70.53 and 59.21% of first and re-infection cases had fever durations of 3–5 and 0–2 days, respectively. Whereas 47.91 and 46.40% of first and re-infection cases had a disease course of 7–9 and 4–6 days.

**Conclusion:**

The SARS-CoV-2 infected individuals in Chengdu, China, during the second pandemic of COVID-19 had mild clinical symptoms and a short course of disease. Furthermore, compared with the first infection, re-infection cases had mild symptoms, low incidences of complications, short fever duration, and course of disease.

## 1 Introduction

After the adjustments to the pandemic prevention policy, China experienced its first COVID-19 (coronavirus disease of 2019) pandemic in December 2022. The primary pandemic strain was Omicron, which has high transmissibility and immune evading capability (1, 2), making it more efficient for re-infection (3). An Italian study compared the incidence of COVID-19 among healthcare workers during the three Severe Acute Respiratory Syndrome Coronavirus 2 (SARS-CoV-2) epidemics, and 98% of the research population received two doses of COVID-19 vaccine during the third epidemic, and the incidence of COVID-19 decreased compared with the second wave (4). A study in Hong Kong showed that although >70% of the population have been vaccinated, the vaccination rate of older adults over 65 years old was relatively low (42%), which leads to a high infection rate of COVID-19 and a high mortality rate in the 5th Wave, and most of the deaths occur among unvaccinated older adults (5). However, the high COVID-19 vaccination rate among vulnerable Singaporeans made the mortality rate much lower during the epidemic of Omicron (6, 7). Some scholars have studied the breakthrough infection after COVID-19 vaccination and showed that 41.5% (391/942) of the subjects were infected with SARS-CoV-2 (8).

Since late April 2023, the number of COVID-19 cases in China has increased, suggesting the start of a second pandemic. However, distinguishing the first infection cases from re-infection cases, as well as epidemiological and clinical characteristics is difficult. Because of the vaccinations and SARS-CoV-2 mutation, re-infection has an impact on the epidemiological modeling of COVID-19 transmission (9, 10). In this study, epidemiological and clinical characteristics of SARS-CoV-2 infected people during the second pandemic of COVID-19 in Chengdu, China were investigated by anonymous questionnaire survey. Furthermore, the differences between first infection and re-infection cases were also compared and analyzed to provide evidence for better prevention and control of SARS-CoV-2 re-infection.

## 2 Subjects and method

### 2.1 Subjects

This study selected people infected with SARS-CoV-2 in Chengdu, China from May 20, 2023–September 12, 2023.

### 2.2 Method

This investigation employed an online platform^1^ to collect the relevant data using an anonymous questionnaire survey. The survey was conducted by forwarding the questionnaire in public through WeChat, posting it in the fever clinic, and pushing its QR code in the hospital follow-up system. The questionnaire included single-and multiple-choice questions, including basic data of SARS-CoV-2 infected people, risk factors of severe illness, vaccination status, clinical manifestation, and treatment. Furthermore, the questionnaire was divided into two parts, where part one comprised questions related to infection and were to be filled by COVID-19 patients during the infection. The second part was only for re-infected patients and was based on questions about their first infection with SARS-CoV-2. The questionnaire was answered by the respondents based on a unified guide language and immediately submitted after acquisition to ensure its validity and authenticity. The Cronbach’s α coefficient of this questionnaire was 0.936, and the KMO was 0.958, *p < 0.05*, indicating good validity and reliability.

For the diagnosis and identification of the population at high risk of COVID-19 severe/critical illness, the Diagnosis and Treatment Protocol for COVID-19 (Trial 10th edition) was referred (11). Cluster infection was defined according to Guidelines for Prevention and Control of COVID-19 (9th edition) (12). The definition of re-infection with SARS-CoV-2 has not yet been agreed upon at home and abroad. In this paper, SARS-CoV-2 re-infection was defined as the recurrence of infection after the first infection has been cured.

Sample size determination: According to the questionnaire design principle and statistical analysis requirements, during estimation, the sample size should be 10–20 times the variable number. This survey was selected 20 times, and the sample size = the maximum number of questions 35*20 = 700. Based on 80% efficiency, a sample size of at least 875 people was acquired.

Sampling procedure: this survey adopts the convenient sampling method.

### 2.3 Statistical method

Statistical analyses were performed using IBM SPSS Statistics Version 22 [Licensed Materials. Property of IBM Corp. ^©^Copyright IBM Corporation and other(s) 1989, 2013]. The normally distributed or approximately normally distributed quantitative data were subjected to a normality test and then expressed as *x̅ ± s.* For intergroup comparisons, the t-test was performed, while for comparing multiple groups, a one-way analysis of variance (ANOVA) was conducted. Not normally distributed quantitative data were expressed as *M* (*P_25_ ~ P_75_*), and their intergroup comparisons were made using the rank-sum test. Count data were expressed as *n* (%), and subjected to *X^2^* test. Differences were considered statistically significant at *p* < 0.05.

## 3 Results

### 3.1 Epidemiological and clinical characteristics of people infected with SARS-CoV-2 during the second pandemic of COVID-19 in Chengdu, China

#### 3.1.1 General information

A total of 1,001 valid questionnaires were received for the first part and the majority of respondents were female [630 (62.94%)], primarily aged between 18–30 [330 (32.97%)], followed by 31–40 (21.58%). Most females [315 (31.47%)] were enterprise workers. Furthermore, 577 (57.64%) of the participants had cluster infection, 74.43% (715) of them received three COVID-19 vaccinations, 7.19% (72) did not receive COVID-19 vaccination at all, while 17.18% (172) did not complete full COVID-19 vaccination course. Moreover, 326 (32.57%) participants were in the severe/critical risk group, 221 (22.08%) had underlying diseases, with cardiovascular and cerebrovascular diseases (including hypertension) being the most prevalent [135 (13.49%)], followed by diabetes [42 (4.2%)].

#### 3.1.2 Clinical manifestation

During the survey, 153 (15.28%) SARS-CoV-2 infected participants did not have a fever, while the remaining had low to moderate fever (Table 1). The duration of fever in the fever group was on average 1–3 days, 663 (78.18%). Other major clinical manifestations included fatigue (*n* = 868; 86.71%), headache (*n* = 756; 75.52%), cough and sputum (*n* = 764; 76.32%), dry throat and sore throat (*n* = 754; 75.32%), muscle soreness (*n* = 693; 69.23%), hypogeusia (*n* = 173; 17.28%), and hyposmia (*n* = 160; 15.98%). Primarily, the symptoms were mild to moderate (Table 2) and the average course of disease duration was 4–6 days (*n* = 288; 37.84%); however, 377 people (49.54%) had a duration of less than 6 days.

**Table 1:** The degree of fever of people infected with SARS-CoV-2 during the second pandemic of COVID-19.

The degree of fever	No fever (1 point)	Low fever (2 point)	Moderate fever (3 point)	High fever (4 point)	Ultra-high Fever (5 point)	*M* (*P_25_* ~ *P_75_*)
*N* (%)	153 (15.28%)	361 (36.60%)	394 (39.36%)	92 (9.19%)	1 (0.10%)	2(2–3)

The above scores are assigned according to the Likert method, where 1 = no fever, 2 = low fever, 3 = moderate fever, 4 = high fever, and 5 = ultra-high fever.

**Table 2:** Other clinical manifestations of people infected with SARS-CoV-2 during the second pandemic of COVID-19.

Clinical manifestation	No symptoms (1 point)	Mild symptoms (2 point)	Moderate symptoms (3 point)	Severe symptoms (4 point)	*M* (*P_25_* ~ *P_75_*)
Weakness	133 (13.29%)	401 (40.06%)	354 (35.36%)	113 (11.29%)	2 (2–3)
Cough and expectoration	237 (23.68%)	388 (38.76%)	288 (28.77%)	88 (8.79%)	2 (2–3)
Headache	245 (24.48%)	452 (45.15%)	259 (25.87%)	45 (4.50%)	2 (2–3)
Sore throat and dry throat	247 (24.68%)	312 (31.17%)	303 (30.27%)	139 (13.89%)	2 (2–3)
Muscle soreness	308 (30.77%)	318 (31.77%)	289 (28.87%)	86 (8.59%)	2 (1–3)
Nasal congestion	544 (54.35%)	269 (26.87%)	146 (14.59%)	42 (4.20%)	1 (1–2)
Runny nose	553 (55.24%)	292 (29.17%)	123 (12.29%)	33 (3.30%)	1 (1–2)
Chest tightness and shortness of breath	748 (74.73%)	190 (18.98%)	51 (5.09%)	12 (1.20%)	1 (1–1)
Hypogustia	828 (82.72%)	107 (10.69%)	48 (4.80%)	18 (1.80%)	1 (1–1)
Olfactory decline	841 (84.02%)	97 (9.69%)	41 (4.10%)	22 (2.20%)	1 (1–1)
Nausea and vomiting	852 (85.11%)	110 (10.99%)	32 (3.20%)	7 (0.70%)	1 (1–1)
Diarrhea	862 (86.11%)	105 (10.49%)	27 (2.70%)	7 (0.70%)	1 (1–1)
Abdominal pain	913 (91.21%)	67 (6.69%)	17 (1.70%)	4 (0.40%)	1 (1–1)
Conjunctival congestion	929 (92.81%)	51 (5.09%)	18 (1.80%)	3 (0.30%)	1 (1–1)

The above scores are assigned according to the Likert method, where 1 = no symptoms, 2 = mild symptoms, 3 = moderate symptoms, 4 = severe symptoms.

#### 3.1.3 Treatment

Among the surveyed population, 726 (72.53%) patients sought medical treatment in hospitals, of which 40 were hospitalized. Furthermore, 249 (76.38%) participants were at high risk of severe/critical disease and sought medical treatment in hospitals, 165 (50.61%) used antiviral medication (including Azvudine, Molnupiravir, and Paxlovid), and 27 (8.28%) were hospitalized for treatment. Of those who were treated in hospitals, 162 (65.06%) were given antiviral medication.

### 3.2 Epidemiological and clinical characteristics of SARS-CoV-2 re-infected people during the second pandemic of COVID-19

#### 3.2.1 People re-infected with SARS-CoV-2

A total of 407 valid questionnaires were received for the second part, indicating that 40.66% (407/1001) of participants were re-infected with SARS-CoV-2. The re-infected participants suffered from the first infection around 5 months before [297 (72.97%)]. Furthermore, the re-infected participants were predominantly females [300 (73.71%)] mostly between 18–30 years old [154 (37.84%)], mostly healthcare and enterprise workers [132 (32.42%) and 112 (27.52%), respectively], and about 253 (62.16%) of these had cluster infection. Moreover, among the re-infected participants, 75.18% (*n* = 306) received three COVID-19 vaccinations, while 5.41% (*n* = 22) did not receive COVID-19 vaccination at all. Additionally, 35 (8.6%) patients were severely obese, and 70 (22.08%) suffered from underlying diseases, the most frequent [39 (9.58%)] being cardiovascular and cerebrovascular diseases (including hypertension), followed by diabetes [13 (3.19%)]. In the re-infection group, 244 people (59.95%) sought medical treatment in hospitals, 7 (1.72%) were hospitalized for treatment, and 59 (14.50%) were taking antiviral drugs. However, in the first infection, only 10 people (2.46%) were hospitalized.

#### 3.2.2 Differences in clinical manifestation of first infection and re-infection with SARS-CoV-2

Of the 407 re-infection cases reported that during their first infection, 20 (4.91%) indicated no fever and if present was moderate to high. Whereas, during re-infection, 105 (25.80%) patients had no fever, while the remaining experienced low to moderate fever (Table 3), and the difference in the degree of fever between the two infections was statistically significant (*p* < 0.05). Furthermore, 27 patients who completed the questionnaire reported that they still had recurrent fever. The analysis of the 380 patients whose temperatures had been completely normalized showed that the re-infection fever duration was on average 0–2 days (*n* = 268; 70.53%), while the first infection fever duration was mostly 3–5 days (*n* = 241; 59.21%) (Table 4). The other important clinical manifestations of the two infections were the same; however, the first infection symptoms were more severe than the re-infection, and the majority of symptoms were statistically different in severity (*p* < 0.05) (Table 5).

**Table 3:** The difference in the degree of fever between first infection and re-infection with SARS-CoV-2.

The degree of fever	No fever (1 point)	Low fever (2 point)	Moderate fever (3 point)	High fever (4 point)	Ultra-high fever (5 point)	*M* (*P_25_* ~ *P_75_*)	*Z*	*P*
First infection	20 (4.91%)	52 (12.78%)	255 (62.65%)	78 (19.16)	2 (0.49%)	3 (3–3)	−15.1	<0.05
Re-infection	105 (25.80%)	187 (45.95%)	100 (24.57%)	14 (3.44%)	1 (0.25%)	2 (1–5)		

The above scores are assigned according to the Likert method, where 1 = no fever, 2 = low fever, 3 = moderate fever, 4 = high fever, and 5 = ultra-high fever.

**Table 4:** The difference in the duration of fever between first infection and re-infection with SARS-CoV-2.

Duration of fever	0–2 d	3–5 d	6–8 d	> 8 d	Total
First infection	118 (28.99%)	241 (59.21%)	33 (8.10%)	16 (3.93%)	407 (100.00%)
Re-infection	268 (70.53%)	92 (24.21%)	13 (3.42%)	7 (1.84%)	380 (100.00%)

**Table 5:** The differences in other clinical manifestations between first infection and re-infection with SARS-CoV-2.

Clinical manifestation	No symptoms (1 point)		Mild symptoms (2 point)		Moderate symptoms (3 point)		Severe symptoms (4 point)		First infection *M (P_25_ ~ P_75_)*	Reinfection *M (P_25_ ~ P_75_)*	*Z*	*P*
	First infection	Re-infection	First infection	Re-infection	First infection	Re-infection	First infection	Re-infection				
Weakness	15 (3.69%)	66 (16.22%)	44 (10.81%)	222 (54.55%)	218 (53.56%)	100 (24.57%)	130 (31.94%)	19 (4.67%)	3 (3–4)	2 (2–3)	−16.00	<0.05
Cough and expectoration	21 (5.16%)	104 (25.55%)	81 (19.9%)	187 (45.95%)	210 (51.6%)	91 (22.36%)	95 (23.34%)	25 (6.14%)	3 (2–3)	2 (1–3)	−13.28	<0.05
Sore throat and dry throat	26 (6.39%)	77 (18.92%)	61 (14.99%)	180 (44.23%)	153 (37.59%)	117 (28.75%)	167 (41.03%)	33 (8.11%)	3 (3–4)	2 (2–3)	−13.00	<0.05
Muscle soreness	38 (9.34%)	151 (37.1%)	70 (17.2%)	186 (45.7%)	194 (47.67%)	55 (13.51%)	105 (25.8%)	15 (3.69%)	3 (2–4)	2 (1–2)	−15.44	<0.05
Headache	45 (11.06%)	106 (26.04%)	117 (28.75%)	201 (49.39%)	165 (40.54%)	86 (21.13%)	80 (19.66%)	14 (3.44%)	3 (2–3)	2 (1–2)	−10.50	<0.05
Nasal congestion	92 (22.6%)	163 (40.05%)	102 (25.06%)	144 (35.38%)	128 (31.45%)	75 (18.43%)	85 (20.88%)	25 (6.14%)	3 (2–3)	2 (1–2)	−8.15	<0.05
Runny nose	107 (26.29%)	166 (40.79%)	116 (28.5%)	156 (38.33%)	129 (31.7%)	64 (15.72%)	55 (13.51%)	21 (5.16%)	2 (1–3)	2 (1–2)	−6.82	<0.05
Chest tightness and shortness of breath	224 (55.04%)	303 (74.45%)	103 (25.31%)	84 (20.64%)	57 (14%)	16 (3.93%)	23 (5.65%)	4 (0.98%)	1 (1–2)	1 (1–2)	−6.56	<0.05
Hypogustia	265 (65.11%)	320 (78.62%)	50 (12.29%)	58 (14.25%)	61 (14.99%)	24 (5.9%)	31 (7.62%)	5 (1.23%)	1 (1–2)	1 (1–1)	−5.04	<0.05
Olfactory decline	272 (66.83%)	334 (82.06%)	50 (12.29%)	43 (10.57%)	57 (14%)	23 (5.65%)	28 (6.88%)	7 (1.72%)	1 (1–2)	1 (1–1)	−5.39	<0.05
Nausea and vomiting	306 (75.18%)	354 (86.98%)	81 (19.9%)	42 (10.32%)	16 (3.93%)	9 (2.21%)	4 (0.98%)	2 (0.49%)	1 (1–1)	1 (1–1)	−4.27	<0.05
Diarrhea	335 (82.31%)	347 (85.26%)	50 (12.29%)	43 (10.57%)	19 (4.67%)	15 (3.69%)	3 (0.74%)	2 (0.49%)	1 (1–1)	1 (1–1)	−1.16	>0.05
Abdominal pain	344 (84.52%)	366 (89.93%)	46 (11.3%)	32 (7.86%)	14 (3.44%)	8 (1.97%)	3 (0.74%)	1 (0.25%)	1 (1–1)	1 (1–1)	−2.34	<0.05
Conjunctival congestion	373 (91.65%)	369 (90.66%)	25 (6.14%)	31 (7.62%)	8 (1.97%)	6 (1.47%)	1 (0.25%)	1 (0.25%)	1 (1–1)	1 (1–1)	−0.46	>0.05

The above scores are assigned according to the Likert method, where 1 = no symptoms, 2 = mild symptoms, 3 = moderate symptoms, 4 = severe symptoms.

#### 3.2.3 Course of disease of first infection and re-infection cases

Among the 407 re-infected participants, the course of disease in the first infection cases was 7–9 days (*n* = 195, 47.91%), while 278 re-infected participants who had recovered reported that the course of disease duration at the time of re-infection was 4–6 days (*n* = 129, 46.40%) (Table 6).

**Table 6:** The difference in the course of disease between first infection and re-infection with SARS-CoV-2.

Course of disease	1–3 d	4–6 d	7–9 d	> 9d	Total
First infection	13 (3.19%)	43 (10.57%)	195 (47.91%)	156 (38.33%)	407 (100.00%)
Re-infection	54 (19.42%)	129 (46.40%)	57 (20.50%)	38 (13.67%)	278 (100.00%)

## 4 Discussion

During the second pandemic of COVID-19 in Chengdu, China, people infected and re-infected with SARS-CoV-2 were predominantly female (*n* = 630; 62.94% and *n* = 300; 73.71%, respectively). This may be because women are responsible for more household activities than men, such as shopping, cleaning, etc., especially caring for sick family members, which increases their risk of infection. Most of the people infected or re-infected with SARS-CoV-2 during the second pandemic were aged 18–30 years (*n* = 330; 32.97% and *n* = 154; 37.84%, respectively), which was consistent with the findings of Eythorsson et al. (13). This may be due to the high mobility of this young population, which increases their risk of SARS-CoV-2 exposure due to more social activities and work interactions. Previous literature suggests that females and younger age people are at a higher risk factors of re-infection (14). Furthermore, this age group of women is more willing to participate in the investigation and since this is a questionnaire-based study, this cohort predominates. Additionally, during the current pandemic, 57.64% of infected people had cluster infections, and 10.49% were not sure if they had cluster infections. These results suggest that good personal protection measures should be taken during the pandemic and gatherings during COVID-19 pandemic season should be avoided to reduce the risk of infection. In this survey, 929 people (92.81%) who were infected with COVID-19 were vaccinated with at least one dose of COVID-19 vaccine, and 715 people (74.43%) were vaccinated with three doses. Therefore, the persistence and effectiveness of the immune response to the COVID-19 vaccine should also be considered. Many studies have found that the IgG antibody in COVID-19 dropped significantly 6 months after COVID-19 vaccination (15, 16). Even studies have shown that the level of COVID-19 IgG antibody in patients dropped by 15 times five months after vaccination (17). Several studies have found that after the completion of the whole course of vaccination, its effectiveness in preventing COVID-19 infection gradually decreases with time; however, its effectiveness in preventing severe illness and hospitalization remains high (90%) (18). Studies have shown that each additional dose of the COVID-19 vaccine reduces the risk of breakthrough infection by 18% and the risk of hospitalization by 25% (19). Moreover, vaccination can also effectively reduce disease severity in those who have been re-infected by COVID-19 (20). Therefore, although the COVID-19 vaccine cannot prevent infection or reinfection 100%, it can effectively reduce the occurrence of severe diseases, thus reducing the disease burden. Thus, it is necessary to strengthen the COVID-19 vaccination and further improve the whole-course immunization, and strengthen the immunization coverage to reduce the occurrence of breakthrough infections and severe infections.

The common COVID-19 clinical symptoms during the current COVID-19 pandemic, include fever, cough and sputum, headache, fatigue, dry throat and sore throat, muscle soreness, etc., consistent with the symptoms observed in the first pandemic in China (21). However, this time, the symptoms were mild to moderate, the fever duration on average was 1–3 days (*n* = 663, 78.18%), and the course of disease duration was <6 days in 377 patients (49.54%), which might be related to the persistent variation of SARS-CoV-2. According to the survey, 162 (65.06%) people were at high risk of severe/critical illness in hospitals and were taking antiviral drugs, indicating that a few individuals (34.94%) at high disease severity risk were not taking antiviral treatment. This might be because of the high cost of antiviral drugs, side effects, or lack of proper knowledge. Early antiviral treatment can effectively reduce the rate of severe illness and mortality (22); therefore, more public education should be provided, the research should be continued, and new antiviral drugs that are cheaper, more effective, and have reduced side effects should be developed.

At the early stage of the pandemic, it was not clear whether re-infection with SARS-CoV-2 would occur until the first case was reported in Hong Kong in April 2020 (23). Subsequently, more and more countries, including the United States (24) and Italy (25) started reporting cases of re-infection. A meta-analysis found that the shortest interval between re-infection and first infection was 19 days, while the longest was 293 days (8). According to Xx et al., the majority of re-infection cases occurred 9 months after the first infection (46.4%) (26). However, some studies suggest that the majority of re-infection cases occur >12 months later (46.8%) (27). In this investigation, the majority of re-infection cases occurred around 5 months (72.97%), which was relatively shorter. This might be because of the adjustment of the pandemic prevention policy, or due to the high population density, mobility, or different susceptibility status in China. Furthermore, it has been indicated that the underlying diseases of re-infected people are not common (28, 29). Here, it was revealed that 82.20% of the re-infected people did not have any underlying diseases, while those with underlying diseases mainly had cardiovascular and cerebrovascular diseases (*n* = 39, 9.58%). Moreover, obesity was also observed in many SARS-CoV-2 re-infected people (*n* = 35, 8.60%), consistent with previous studies (30).

In this investigation, the main clinical manifestation of SARS-CoV-2 re-infected patients was the same as the first infection; however, the re-infection symptoms were milder than the first infection, and the incidence of each symptom was lower, in line with the conclusions of previous studies (31, 32). The fever duration of re-infection was also shorter than that of the first infection. Among the re-infected people, 244 (59.95%) visited hospitals, which is inconsistent with the findings of Chen et al. (31), who concluded that only 9.6% of the re-infected people visited outpatient and emergency departments of healthcare institutions. This may be because the questionnaire QR code was posted at the fever clinic of the hospital and was pushed using the follow-up system of the hospital, which may have skewed the data and may also be related to the different concepts of patient care. An observational study in Serbia revealed that 99.17% of SARS-CoV-2 re-infected people had mild symptoms, a hospitalization rate of 1.08%, and a mortality rate of 0.15% (33). Whereas, the duration course of the first infection was 7–9 days (*n* = 195, 47.91%), with 351 people (86.24%) indicating a period of 7 days and above, and the duration of the re-infection was on average 4–6 days (*n* = 129, 46.40%), while 183 (63.76%) patients had a course of 6 days or less. This indicated that the course of re-infection was shorter, consistent with the previous studies (31), suggesting that the pathogenicity of COVID-19 is decreasing with the continuous mutation of SARS-CoV-2 and COVID-19 vaccination. Moreover, Flacco ME et al. concluded that the overall severe illness/death rate after re-infection was very low (2/10000–7/10000) (34).

## 5 Conclusion

COVID-19 vaccination can effectively reduce the occurrence of breakthrough infections and severe cases, therefore, it is necessary to continue to improve the coverage of whole-course immunization and strengthen immunization in China. Studies have indicated that most people (60%) are willing for simultaneous administration of COVID-19 vaccine booster and influenza vaccine (35). Simultaneous vaccination is important, it neither affects the safety of products nor increases the risk of breakthrough infection in COVID-19 (8). Currently, both COVID-19 and influenza vaccines are accessible in China. At the national level, publicity can be strengthened through various ways to enhance people’s awareness of the prevention knowledge of respiratory infectious diseases and their recognition of vaccines. People should be vaccinated before the epidemic season of respiratory infectious diseases, and should also be given personal protection at the same time, especially those who are at a high risk of disease severity to reduce the occurrence of re-infection. Furthermore, doctors should be consulted timely upon observing COVID-19 symptoms, and if necessary, antiviral medication should be taken under the guidance of professionals, as early as possible.

## Data availability statement

The datasets presented in this study can be found in online repositories. The names of the repository/repositories and accession number(s) can be found in the article/supplementary material.

## Ethics statement

The studies involving humans were approved by Medical Ethics Committee of The Third People’s Hospital of Chengdu. The studies were conducted in accordance with the local legislation and institutional requirements. Written informed consent for participation in this study was provided by the participants’ legal guardians/next of kin.

## Author contributions

CL: Data curation, Formal analysis, Investigation, Methodology, Writing – original draft. TZ: Writing – original draft, Investigation, Methodology. PZ: Writing – review & editing. JH: Writing – review & editing. YL: Writing – review & editing.

## Footnotes

^1^ https://www.wjx.cn
